# Compression Stocking Length Effects on Oedema, Pain, and Satisfaction in Nursing Students: A Pilot Randomized Trial

**DOI:** 10.3390/healthcare8020149

**Published:** 2020-05-29

**Authors:** Yoonyoung Lee, Kisook Kim, Seunghyun Kang, Ji yeong Kim, Su gyeong Kim, Taeun Kim, Jisu Jung

**Affiliations:** 1 Department of Nursing, Sunchon National University, Suncheon, Jeollanam-do 57922, Korea; yoonyoung1@hanmail.net (Y.L.); by6963@naver.com (S.g.K.); 2 College of Nursing, Chung-Ang University, Seoul 06974, Korea; 3 Department of Nursing, Korea University Ansan Medicine, Ansan, Geyonggi-do 15355, Korea; rkd1275@naver.com; 4 Department of Nursing, Kyunghee University Medical Center, Seoul 02447, Korea; wldud7983@naver.com; 5 Army Nurse, Armed Forces Medical School, Daejeon 34059, Korea; ktw2406@naver.com; 6 Department of Nursing, Asan Medical Center, Seoul 05505, Korea; jungga333@gmail.com

**Keywords:** compression stockings, oedema, pain, satisfaction, nursing students

## Abstract

Professional practitioners who are required to stand for long periods of time frequently complain about vein-related symptoms. Compression stocking are effective for vein-related symptoms, but there is not enough evidence on the effect of the length of compression stockings for nursing students. To compare oedema, pain, and satisfaction according to different lengths of compression stockings worn by female nursing students. This study was conducted as a randomized clinical trial. The participants included 20 female nursing students in their first semester of clinical practice training. Compression stockings with 25–32 mmHg pressure were used in the study; the participants were divided into two groups based on the length of compression stocking: knee length and thigh length. Differences between groups regarding pain, oedema, and satisfaction were analysed using t-tests, paired t-tests, and Mann–Whitney U tests, when appropriate. There were no significant differences in pain, oedema, and satisfaction between the two groups. However, pain in right legs of the thigh-length stocking group significantly increased after clinical training shift compared with that before the shift (t = −2.377, *p* = 0.041). Both groups reported high satisfaction. There were no differences in pain, oedema, and satisfaction in both legs based on the length of compression stockings, but side effects appeared in participants wearing the thigh-length stockings; nevertheless, satisfaction was high in both groups. It may be important to suggest nursing students to wear knee-length compression stockings during clinical practice training.

## 1. Introduction

Within the general population at large, the experience of heavy legs and increased pain from oedema after sitting or standing all day is common [1,2,3]. Professional practitioners who are requiredto stand for long periods of time frequently complain about vein-related symptoms (e.g., leg pain, oedema, and heaviness of the legs) [4]. In particular, nurses often complain of lower extremity oedema and lower back pain after work [5]. According to one study, 97% of nursing students experience lower extremity pain, 87% report lower extremity oedema, and 30% report both severe lower extremity oedema and pain [6].

To address this problem, compression therapy is often used to resolve insufficient lymphatic and venous circulation in the lower limbs [7]. Compression stockings [8], pneumatic devices [9], elastic and non-elastic bandages, boots, and hosiery have been used for compression therapy [7]. Graduated compression stockings are a non-invasive standardized treatment for venous and lymphatic diseases [3]. Compression stocking work by maximizing pressure in the ankle and gradually compress over the stockings so that blood flows towards the heart. Compression stockings reverse venous blood hypertension, increase skeletal muscle pumps, promote blood return to the veins, and improve lymphatic drainage [7]. Several studies have reported that compression stockings with pressures of approximately 25–30 mmHg were near effective in reducing leg oedema [10,11]. However, studies specifically examining the side effects of compression stockings are lacking [12,13]; the use of stockings can cause discomfort, pressure necrosis, ischaemia, contact dermatitis, skin discoloration, and blistering [14].

Various studies have been conducted to examine the effects of using compression stockings. A randomized controlled trial including the use of compression stockings in healthy adult women reported that their lymphatic pumping pressure was improved [11]. Additionally, another randomized crossover trial on compression hosiery worn by hairdressers reported reduced discomfort, oedema, and pain in the legs [4]. Another study compared the use of compression stockings with non-compression stockings in patients with varicose vein symptoms and demonstrated that compression stockings were effective for pain but not for oedema or paraesthesia [15]. On the basis of these experimental studies, systematic reviews have been conducted on the effects of compression stockings on oedema risk and pain in healthy adults, which include the prevention and treatment of chronic venous disease, varicose ulcers, and deep venous thrombosis and treatment of lymphoedema [3]. One study has shown that the use of compression stockings in nursing students during clinical practice training is very effective at reducing oedema and pain [6]. However, that study was not a randomized controlled study, did not account for differences in activity levels during the students’ practice, and included non-homogeneous participants.

Other studies have compared pressure intensity [16,17], with the majority comparing pressure stockings with placebo stockings [3,15]. Most studies incorporating different lengths of compression stockings examined the prevention of deep vein thrombosis. In one study, there were no differences in the prevention of thrombus according to stocking length [18]. Regarding studies examining ways to prevent deep vein thrombosis, a systematic review showed that thigh-length stockings are preferred and more effective [19]. There have been systematic literature reviews suggesting that thigh-length stockings may be more effective than knee-length stockings; however, well-conducted randomized clinical trials are lacking [19,20].

Nurses and nursing students often experience pain and oedema of the lower limbs such as occupational oedema because of prolonged standing or sitting time [6,10]. Therefore, nursing students should know how compression stockings may affect them and select the appropriate stocking length accordingly. Scientific evidence should be provided through well-conducted randomized clinical trials. In addition, the degree of oedema and pain in the lower extremities can differ between women and men [21]. This pilot study intended to inform many female nursing students on the effect of compression stockings. However, most studies showed inconsistent findings compared with the results of this study on the length of compression stockings. Additionally, previous studies did not examine the effects on lower extremity pain and oedema in nursing students, and existing randomized clinical trials have not reported generalization of the findings that could be applied to nursing students.

Therefore, the purpose of this study was to examine oedema, pain, satisfaction, and side effects with compression stockings of different lengths worn by female nursing students. This study will be used as the groundwork for additional research, confirming the effects of compression stockings of various lengths and materials and further developing tools for pain and oedema of the lower extremities.

## 2. Participants, Ethics, and Methods

### 2.1. Design

This study was conducted as a randomized clinical trial.

### 2.2. Participants

The participants of this study were female nursing students in their first semester of clinical training. Inclusion criteria were female nursing students who received explanation and consented to the study, and who were performing day or evening shift clinical practice in the general ward during their first clinical practice semester.

The exclusion criteria were any evidence of oedema or pain during clinical practice training and skin diseases including wounds on the feet or lower limbs. Further exclusion criteria were any medical records on history of heart, liver, kidney, and vascular diseases. Participants were also required to understand the purpose of this study and have a body mass index within the normal range. Those with prior experience of wearing compression stockings were also excluded. A final sample of 20 participants was included in the study; they were randomly assigned to either of the stocking length groups. No participants dropped out of the study. Since study participants may experience different degrees of pain and oedema according to their activity level [22], any differences between the groups were examined by checking the number of steps when the stockings were applied.

#### 2.2.1. Randomization and Blinding

Participants were randomly assigned to each group by using a computer-generated randomized number table. The group assigned to each participant was placed in a sealed envelope and distributed to research assistant A. Following this, research assistant B measured pain, oedema, and satisfaction without knowing which group the participant belonged to. Research assistant B also measured pain and oedema without the elastic compression stocking 10 min before the participant started clinical training and measured pain, oedema, and satisfaction after removing the elastic compression stocking 10 min after the end of the clinical training. Although the study included randomization of each participant, they were not blinded to the two interventions.

#### 2.2.2. Intervention

Data were collected from 5 November to 14 December 2018 at the clinical practice hospital in Suncheon, South Korea. The compression stockings used in the study (Jobstocking®, Calze Olona, Varesino, Italy) were made of Lycra and polyamide. The two compression stockings were all-the-toe-exposed type. The pressure of the knee-length compression stockings was 25–32 mmHg in the ankle and 18–22 mmHg in the calf. The pressure of the thigh-length compression stockings was 25–32 mmHg in the ankle, 18–22 mmHg in the calf, and 10–13 mmHg in the thigh. In this study, group 1 was given knee-length elastic compression stockings and group 2 was given the thigh-length elastic compression stockings for a duration of 9 h each during the participant’s clinical training shift.

### 2.3. Outcome Measurements

All data were recorded by research assistant B. Pain was measured by a numeric rating scale (NRS) and measured 10 min before and after participant’s clinical training. Oedema was marked with an indelible pen at a point 10 cm below the tibial tuberosity, and the research assistant measured the value expressed in centimetres using a glass fibre tape measure (200 cm, Hwashin^®^, Paju, Korea). Oedema was also measured 10 min before and 10 min after participant’s clinical training. Satisfaction was measured by an NRS following the participant’s clinical training shift. The general characteristics were investigated after clinical training. The side effects of applying compression stockings were open-ended questions of peripheral neurosis, skin damage to the bone protrusion, dermatitis, and allergic reactions to the fabric.

### 2.4. Ethical Considerations

This study was approved by institutional review board (IRB) (1040173-201807-HR-017-06) at the Bioethics Review Committee of the institute affiliated with the researcher and registered with the Clinical Research Information Service (KCT0003768). After the IRB approval, participants were selected from those who agreed to the study after receiving explanations about the research process, study period, and precautions. Compression stockings were provided to the study participants after obtaining consent.

### 2.5. Data Analysis

The data were analysed following the intention-to-treat principle. Using SPSS 23.0 (IBM Corp., Armonk, NY, USA), general participant characteristics were analysed using descriptive statistics. The normality of continuous variables was confirmed using the Shapiro–Wilk test. The height, weight, activity, pain, oedema, and satisfaction scores were confirmed to be normally distributed and were analysed using *t*-tests. The test for normality was not significant for age (knee-length stockings W = 0.507 *p* = 0.010; thigh-length stockings W = 0.614 *p* = 0.010) and, therefore, analyses were conducted using the Mann–Whitney U test. To examine the differences in pain and oedema before and after wearing compression stockings, paired t-tests were used.

## 3. Results

### 3.1. Characteristics of Subjects and Homogeneity

**Figure 1:**
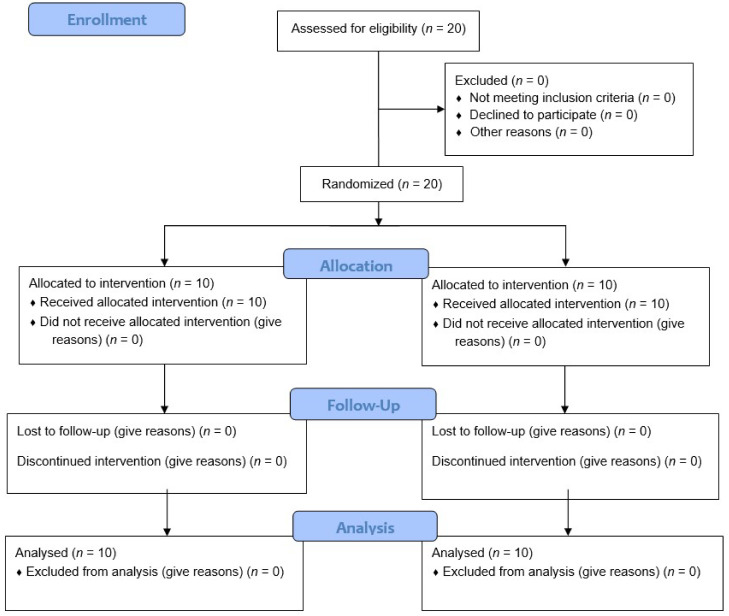
CONSORT Flow Diagram.

Figure 1 shows the CONSORT flow diagram. All 20 subjects were randomly assigned to one of the two groups and were included in the study, whereby no participants were excluded during the study. Table 1 shows general characteristics and the homogeneity of the two groups. There were no differences between groups with respect to age, height, weight, and activity of clinical practice training.

### 3.2. Differences between Groups Based on Compression Stocking Lengths

Table 2 shows differences between groups according to the length of compression stockings. There were no statistically significant differences in pain, oedema, and satisfaction between groups according to the length of compression stockings. However, satisfaction was higher in group 1 (7.3 ± 1.5) than in group 2 (6.8 ± 2.5).

### 3.3. Differences in Pain, Oedema, and Satisfaction before and after Wearing Compression Stockings

Table 3 shows differences in pain and oedema before and after wearing each type of compression stocking. In group 2, pain increased significantly only in the right leg following clinical training (t = −2.377, *p* = 0.041). There were no statistically significant differences in pain in the left and right legs of participants in group 1, although pain increased in the left legs of participants in group 2. Although the difference was not statistically significant, group 1 experienced increased oedema in the right leg. However, decreased oedema was observed in the left leg of group 1 and in both legs of group 2.

### 3.4. Side Effects of Wearing Compression Stockings

When responding to the open-ended questions about the side effects of compression stockings, one subject wearing the thigh-length stockings complained of feelings of tightness and itching.

## 4. Discussion

This study incorporated a randomized test to compare oedema, pain, and satisfaction in female nursing students wearing knee-length and thigh-length compression stockings. There were no statistically significant differences in pain, oedema, and satisfaction according to stocking length. In a previous study conducted to reduce oedema and pain in the legs of students, a convenience sampling method was employed. Studies that incorporate the use of compression stockings in female nursing college students who practice clinically have found reductions in oedema and pain [6] and that foot baths additionally reduce oedema and pain [23]. These studies involving nursing students were limited in generalizability because of the use of convenience sampling.

In the current study, there were no statistically significant differences in pain, oedema, and satisfaction between the two compression stocking length groups. These results were similar to those of a study suggesting no differences in the potential prevention of deep vein thrombosis according to stocking length [18]. However, the current study results differ from the results of systematic literature reviews [19,24] demonstrating that thigh-length stockings were preferred and more effective. Given there were no significant differences between the effects of stocking lengths, it appears that students can choose their preferred length without experiencing disadvantages or greater side effects from one or the other type of stockings.

Pain was not significantly different between groups according to stocking length. However, both knee-length stockings and thigh-length stockings increased leg pain after training, especially in the right leg in the thigh-length stocking group. During clinical placements, it is shown that compression pain to help venous circulation is not reduced due to muscle pain caused by increased activity of the leg muscles. Therefore, additional research related to compression stockings, pain control effects, and pain induction is needed.

There were no statistically significant differences in oedema according to stocking length. However, oedema decreased in the left legs of participants wearing knee-length stockings and in both legs of participants wearing thigh-length stockings after clinical training. These results are similar to those of a study that examined compression stocking wear during a 3 h flight [8]. The main reason to wear compression stocking is to improve venous circulation and reduce oedema, which seems to improve venous circulation regardless of length, even in disease-free students. One systematic literature review [25] demonstrated that compression stockings reduce oedema, although this effect was largely reported by poor-quality studies. This study is considered to be a study that can theoretically support the effect of compression oedema reduction.

In this study, there were no statistically significant differences in satisfaction according to stocking length, but most participants reported high satisfaction overall. Participants wearing knee-length stockings reported higher satisfaction than those wearing thigh-length stockings. This finding conforms to the results of a similar study examining the effect of stocking length on treatment of chronic venous insufficiency [26]. In that study, thigh-length stockings were more effective in preventing venous stagnation than knee-length stockings, and quality of life and satisfaction was higher than that in those with knee-length stockings. Thus, thigh-length stockings seem to cause a lot of discomfort for participants in daily life but are considered better to wear and have higher satisfaction if worn for disease-treatment purposes.

In the current study, one participant wearing thigh-length stockings complained about feelings of tightening and itching. This is consistent with other studies that reported similar side effects when individuals wore compression stockings [14], including discomfort, pressure necrosis, ischaemia, contact dermatitis, skin discoloration, and blistering. It appears that the compression stockings that wrap around the entire leg rather than the knee contribute to discomfort and itching due to ischaemia because of a wider area of contact. Therefore, it is important for nursing students to take these side effects into consideration when choosing stockings.

Nursing students complain of physical challenges including tension, lower extremity oedema, and pain from standing for long periods of time. Additionally, students report psychological anxiety related to unfamiliar hospital environments during their first clinical placement. Similar to a study [6], which claimed to help increase efficiency and satisfaction during training, this study presents the effect of compression stockings on the lower extremities of nursing students with respect to oedema and satisfaction during clinical practice.

The major limitation of this study is the small number of study participants, as it was difficult to recruit participants from a specific population (i.e., nursing students in training). Therefore, additional randomized trials with sufficient numbers of participants are required for greater generalizability of the study results. In addition, male nursing students may also experience leg pain and oedema, but since the participants sampling was limited to female nursing students in this study, studies on male nursing students with sufficient sampling will be needed in future studies.

## 5. Conclusions

In this study, we compared oedema, pain, and satisfaction according to the length of compression stockings worn by female nursing students. There were no statistically significant differences in pain, oedema, and satisfaction reported according to the length of stockings worn, although satisfaction was high in participants wearing knee-length stockings. Discomfort and itching appeared in participants with thigh-length stockings. These findings contribute to the growing literature examining the implications of compression stocking length in clinical settings and other professions where individuals stand for long hours.

## Figures and Tables

**Table 1:** Basic characteristics and homogeneity tests between groups.

Variables	Group 1 (*n* = 10)	Group 2 (*n* = 10)	t or z	*P*
	Median (IQR) or M ± SD	Median (IQR) or M ± SD		
Age (years)	22 (0.75)	22 (1.5)	−0.348 ^†^	0.728
Height (cm)	159.2 ± 5.0	159.9 ± 5.3	−0.282	0.781
Weight (kg)	51.5 ± 5.4	52.0 ± 5.9	−0.199	0.845
BMI (kg/m^2^)	20.3 ± 2.0	20.3 ± 1.8	−0.047	0.963
Activity of training (steps)	7098.9 ± 3356.0	5948.9 ± 3571.7	0.742	0.468

^†^ Mann–Whitney U test; IQR: interquartile range; BMI: body mass index.

**Table 2:** Differences in pain, oedema, and satisfaction between groups according to length of compression stockings.

Variables		Group 1 (*n* = 10)	Group 2 (*n* = 10)	t	*p*
		M ± SD	M ± SD		
Pain of right leg	pre-test	1.2 ± 1.1	1.7 ± 1.5	−0.842	0.411
	post-test	1.9 ± 1.2	2.6 ± 1.3	−1.271	0.220
Pain of left leg	pre-test	1.2 ± 1.1	1.7 ± 1.6	−0.817	0.425
	post-test	1.8 ± 1.1	2.6 ± 1.3	−1.488	0.154
Oedema of right leg (cm)	pre-test	33.5 ± 2.0	34.0 ± 1.8	−0.606	0.552
	post-test	33.7 ± 2.1	33.5 ± 1.9	0.192	0.850
Oedema of left leg (cm)	pre-test	33.6 ± 2.1	33.7 ± 1.8	−0.124	0.902
	post-test	33.4 ± 1.8	33.5 ± 2.0	−0.059	0.954
Satisfaction	post-test	7.3 ± 1.5	6.8 ± 2.5	0.538	0.597

**Table 3:** Mean differences in pain and oedema between stocking lengths before and after participants’ clinical training shifts.

Variables	Group 1 (*n* = 10)	T	*p*	Group 2 (*n* = 10)	T	*p*
	M ± SD			M ± SD		
Pain of right leg	−0.70 ± 1.42	−1.561	0.153	−0.90 ± 1.20	−2.377	0.041 *
Pain of left leg	−0.60 ± 1.43	−1.327	0.217	−0.90 ± 1.45	−1.964	0.081
Oedema of right leg	−0.23 ± 0.79	−0.917	0.383	0.45 ± 1.23	1.159	0.276
Oedema of left leg	0.19 ± 0.61	0.991	0.348	0.25 ± 0.77	1.032	0.329

* *p* > 0.05.
